# Association Between the Loss of Gait Harmony and Cognitive Impairment: Cross-Sectional Study

**DOI:** 10.2196/46264

**Published:** 2023-07-10

**Authors:** Ju-Young Choi, Sang-Won Ha, Da-Eun Jeong, Jaeho Lee, Donghoon Kim, Jin-Young Min, Kyoung-Bok Min

**Affiliations:** 1 Department of Preventive Medicine College of Medicine Seoul National University Seoul Republic of Korea; 2 Department of Neurology Veterans Health Service Medical Center Seoul Republic of Korea; 3 Integrated Major in Innovative Medical Science Seoul National University Graduate School Seoul Republic of Korea; 4 Veterans Medical Research Institute Veterans Health Service Medical Center Seoul Republic of Korea; 5 Institute of Health Policy and Management Seoul National University Medical Research Center Seoul Republic of Korea

**Keywords:** cognitive function, gait phase, physical performance, dementia, older adult, aging, asymmetric, balance, gait analysis, cognition, cognitive impairment, gait, gait pattern, aging

## Abstract

**Background:**

Functional limitations and disabilities have been associated with a decrease in cognitive function due to increasing age. Gait performance and cognitive function have been associated with gait variability in executive function, the phase domain in memory, and gait abnormalities in cognitive decline.

**Objective:**

Our study aimed to investigate whether gait harmony was associated with cognitive function in the older adult population. Moreover, we aimed to investigate whether gait harmony was associated with cognitive function and explore each cognitive function in a specific harmonic state.

**Methods:**

The study population included 510 adults aged ≥60 years who visited the Department of Neurology at the Veterans Health Service Medical Center, Seoul, South Korea. Gait data were collected using a 3D motion capture device with a wireless inertial measurement unit system. For cognitive function assessments, we used the Seoul Neuropsychological Screening Battery-Core test, which evaluates the level of cognitive function or impairment in 5 cognitive domains.

**Results:**

In general, the association between the Seoul Neuropsychological Screening Battery-Core tests and the stance-to-swing ratio in the >1.63 ratio group yielded lower β coefficients than those in the 1.50-1.63 ratio group. After adjustment for confounders, the odds ratio (OR) for the Digit Symbol Coding test (adjusted OR 0.42, 95% CI 0.20-0.88) and the Korean version of the Color Word Stroop Test: 60 seconds (adjusted OR 0.51, 95% CI 0.29-0.89) for frontal and executive function were significantly lower for the >1.63 ratio group than the reference group.

**Conclusions:**

Our findings suggest that the gait phase ratio is a valuable indicator of walking deficits and may also be associated with cognitive impairment in older adults.

## Introduction

The global population is rapidly aging, and the number of people aged 65 years or older is expected to increase from 703 million in 2019 to 1.5 billion in 2050 [1]. With the increasing older adult population, age-related cognitive impairment has increased and will become a public health problem unless prevention and intervention are implemented [2]. The development of many neurodegenerative disorders is usually a feature of the older adult population in the context of aging [3], and age is known as the primary risk factor for many diseases such as Alzheimer disease (AD) and dementia [4,5]. Approximately 50 million people have been diagnosed with dementia, which is one of the leading causes of disability and mortality among aging adults [6]. Moreover, dementia resulting from cognitive decline is an irreversible process, and there are no effective treatments or drugs for dementia [7]. Therefore, it is important to identify factors that can delay the onset of cognitive impairment or impact cognitive outcomes.

Human gait is complicated and divided into 5 primary modal domains: rhythm, phase, variability, pace, and base of support [8]. Safe and effective gaits are important markers throughout life [9]. In older individuals, gait is used as a predictor of health status [10], falls, activities of daily living [11,12], risk of dementia [13], and even risk of early mortality [14]. Functional limitations and disabilities have been associated with a decrease in cognitive function due to increasing age [15]; motor performance may be related to cognitive impairment and dementia [16,17]. Furthermore, impairment of the motor system, such as gait abnormalities, can lead to cognitive decline [16,17] or early stages of dementia with aging [18]. Gait performance and cognitive function have been associated with gait variability in executive function [19], the phase domain in memory [20], and gait abnormalities in cognitive decline [21]. An abnormal gait, which increases the risk of dementia in older adults, suggests low physical fitness in areas such as mobility [22] and balance [23,24]. Therefore, gait cannot be simply explained as an autonomic movement.

A principal issue is that older adults who demonstrate an imbalance in gait are likely to be at a higher risk of cognitive impairment [25]. Gait harmony, which indicates the proportion of stance to swing in kinematic gait, is defined as the capacity to transform the symmetrical human body into alternated, synchronized, symmetric, and rhythmic movements [26]. Previous studies have shown that the proportion of stance to swing is reduced during fast walking and increases under pathological conditions such as Parkinson disease (PD) [27], stroke [28], and spastic cerebral palsy [29]. A ratio from stance to swing of 70% to 30% was found in patients with stroke [28], and a longer stance phase increased gait stability [30]. It has been suggested that a stance between 59% and 70% is a good compromise between fast and stable walking by the harmonic properties of the stance-to-swing ratio [30]. The harmonic ratio property is founded not only in various disparate physical and biological systems, including leaf disposition [31], nucleotide frequencies [32], and cell [33], but also in human sciences, such as body proportions [34] and aesthetic preferences [35]. However, there is a lack of evidence supporting the association between gait harmony and cognitive function.

Therefore, the objective of this study was to investigate whether gait harmony was associated with cognitive function in the older adult population. In addition, we aimed to explore each cognitive function in a specific harmonic state.

## Methods

### Setting and Study Design

A cross-sectional study was conducted at the Veterans Health Service Medical Center, Seoul, South Korea. Individuals aged ≥60 years who visited the Department of Neurology between March and December 2021 were recruited in this study. The inclusion criteria were as follows: (1) those concerned about cognitive decline, (2) those who could independently undergo all the clinical tests and respond to questionnaires, and (3) those who agreed to participate in this study. Individuals were excluded based on the following criteria: (1) a diagnosis of dementia (International Classification of Diseases, 10th Revision, codes F00-F09 and G30); (2) diagnoses of brain infarction, cerebral hemorrhage, or PD; and (3) a diagnosis of another serious disease (eg, cancer or mental illness). Experienced neurological clinicians evaluated the inclusion and exclusion criteria.

The participants were subjected to a health survey consisting of gait measurements, cognitive examinations, and questionnaires. The survey was conducted at the Veterans Medical Research Institute of the Veterans Health Service Medical Center. A total of 575 individuals volunteered to participate in this study and provided informed consent at enrollment. Of these, 65 participants subsequently dropped out of the study because they were younger than 60 years (n=5) or had missing data on variables (ie, sex, education level, BMI, gait measurements, and cognitive parameters; n=60). After these exclusions, 510 people were eligible for this study.

### Ethics Approval

Study protocols were approved by the Institutional Ethics Review Board of the Veterans Health Service Medical Center (BOHUN 2021-02-024 and BOHUN 2021-01-066). All participants provided signed informed consent prior to study enrollment.

### Gait Analysis

Kinematic data of gait parameters were collected using the NORAXON myoMOTION sensor, which is a 3D motion capture device used with a wireless inertial measurement unit (IMU) system. The IMU sensor plays a role in transmitting human movement data directly to the myoMOTION receiver to compute the angular changes of the selected body segments. In a particular space, the 3D rotation angles of each sensor on selected body segments were measured from the 3D accelerometer, gyroscope, and magnetometer using *fusion algorithms*; these angles are also known as orientation or navigation angles. The sensor for angular orientation uses a robust fusion algorithm combining the elemental sensor component axes’ readings as quaternion element values. This technology mathematically combines and filters incoming source signals at the sensor level and transmits the 4 quaternions of each sensor. Then, using NORAXON’s software, the quaternion data are automatically converted into anatomical angles using a rigid body model with joint segments. NORAXON’s software also records each sensor’s orientation angles and linear acceleration. The system is intended to quantify angular changes of the involved joint and can mathematically derive the x, y, and z displacements in space from linear acceleration data and inverse kinematics modeling. Using this process, the algorithm uses a gyroscope and acceleration data from body-mounted sensors to identify the stance and swing phases in the gait cycle through the signal recorded at a sampling rate of 100-200 Hz. The recoded IMU data were added, filtered, and processed using the myoMOTION software to quantify angular changes in the joints, and the output was exported to Microsoft Excel files [36-38].

For this study, 7 IMU sensors were attached to the participants’ feet, frontal tibia bones, quadriceps, and the sacrum on the left and right sides symmetrically (ie, 2 on the feet, 2 on the frontal tibia bones, 2 on the quadriceps, and 1 on the sacrum, respectively). Calibration was performed using an upright posture to determine the value of the 0° angle in the joints. The participants were instructed to walk at their usual pace for the measured distance (10 m), and walking start or stop points for the measured distance were marked on the floor. The participants started walking at the start signal and stopped walking by themselves at the stop point. However, owing to an acceleration and deceleration phase during the 10-m gait test, the first and last steps were removed.

The data transmitted to the myoMOTION software comprised the kinematic parameters of gait variables including the automatically processed average signals for each stance and swing phase. The stance-to-swing ratio was calculated using the average signal values of the stance and swing phases (*stance phase [%] / swing phase [%]*) [30]. The total gait cycle comprises the stance and swing phases. The literature suggests that during stable human walking, with the total gait cycle set to 100%, the stance phase comprises 60% to 62% and the swing phase comprises 40% to 38%, equating to a ratio of 1.50-1.63 [39-41]. These values were then used to determine the harmonic gait group in this study. Therefore, gait ratios were stratified into 3 groups (<1.50, 1.50-1.63, or >1.63). The harmonic group included individuals in the 1.50-1.63 ratio range, whereas the nonharmonic group included those with the other ratios (<1.50 and >1.63).

### Cognition Evaluation

Participants performed a brief version of the Seoul Neuropsychological Screening Battery, named the Seoul Neuropsychological Screening Battery-Core (SNSB-C), which evaluates the level of cognitive function or impairment in the following 5 cognitive domains: attention, language and related functions, visuospatial functions, memory, and frontal and executive functions [42]. SNSB-C is made up of 14 subtests, including the Digit Span Test, a short version of the Korean-Boston Naming Test (S-K-BNT), Rey Complex Figure Test (RCFT), Seoul Verbal Learning Test: Delayed Recall (SVLT: DR), the Korean version of Color Word Stroop Test: 60 seconds (K-CWST: 60sec), Controlled Oral Word Association Test (COWAT), Trail Making Test-Elderly: Part B (TMT-E: B), and Digit Symbol Coding [43]. Each SNSB-C score is expressed as standardized percentile, stratified for age, sex, and education.

### Other Variables

The questionnaire information included age (≥60 years) and sex (male or female). Health behavior variables included smoking status (current, former, or never), alcohol consumption (drinker or nondrinker), vigorous exercises (yes or no), and BMI. BMI was calculated by dividing the individual’s weight (kg) by height squared (m^2^).

### Statistical Analyses

Statistical differences between the characteristics of the study population were analyzed according to the stance-to-swing ratio (<1.50, 1.50-1.63, or >1.63 ratio group) using the proportion of physiologically comfortable human gait [30,40,41,44,45]. In the analysis, cognitive function and gait ratio were used as the dependent and independent variables, respectively. For each variable, the chi-square test and 2-tailed *t* test were performed for each group of participants. Linear regression analysis was used to evaluate the association between cognitive function tests and the stance-to-swing ratio in each group of gait phase ratio and to provide β coefficients and SEs. The logistic regression model provided odds ratios (ORs) and 95% CIs for each cognitive function test percentiles as the stance-to-swing ratio increased or decreased. The 1.50-1.63 ratio group was used as the reference. Regression models were adjusted for age, sex, education level, smoking status, alcohol consumption, vigorous exercises, and BMI. All statistical analyses were performed with SAS (version 9.4; SAS Institute), and the statistical significance was established at α=.05.

## Results

### Participant Characteristics

Table 1 summarizes the characteristics of the study population according to the 3 stance-to-swing ratio groups. A total of 510 participants with a mean age of 74.1 (SD 5.6) years were included in the study. Of the participants, 34 (6.7%) were in the <1.50 ratio group, 122 (23.9%) were in the 1.50-1.63 ratio group, and 354 (69.4%) were in the >1.63 ratio group.

**Table 1:** Characteristics of the study population according to the 3 stance-to-swing ratio groups.

Characteristic		Total population (n=510)	Stance-to-swing ratio group^a^				*P* value^b^	
			<1.50	1.50-1.63	>1.63			
Participant, n (%)		510 (100)	34 (6.7)	122 (23.9)	354 (69.4)			
Age (year), mean (SD)		74.1 (5.6)	74.1 (5.2)	73.7 (5.0)	74.3 (5.8)	.56^c^		
**Sex, n (%)**								.57^d^
	Male	272 (53.3)	17 (6.3)	70 (25.7)	185 (68)			
	Female	238 (46.7)	17 (7.1)	52 (21.9)	169 (71)			
**Education, n (%)**								.06^d^
	Less than high school	259 (50.8)	13 (5)	51 (19.7)	195 (75.3)			
	High school	118 (23.1)	9 (7.6)	34 (28.8)	75 (63.6)			
	College or more	133 (26.1)	12 (9)	37 (27.8)	84 (63.2)			
**Smoking status, n (%)**								.66^d^
	Current or former smoker	196 (38.4)	12 (6.1)	51 (26)	133 (67.9)			
	Never smoker	314 (61.6)	22 (7)	71 (22.6)	221 (70.4)			
**Alcohol drinking, n (%)**								.12^d^
	Drinker	308 (60.4)	18 (5.8)	83 (27)	207 (67.2)			
	Nondrinker	202 (39.6)	16 (7.9)	39 (19.3)	147 (72.8)			
**Vigorous intensity exercises for a week, n (%)**								.045^d^
	Yes	101 (19.8)	12 (11.9)	26 (25.7)	63 (62.4)			
	No	409 (80.2)	22 (5.4)	96 (23.5)	291 (71.2)			
BMI (kg/m^2^), mean (SD)		24.9 (3.2)	24.1 (2.5)	23.9 (2.9)	25.4 (3.2)	<.001		
**Gait phase (%), mean (SD)**								
	Stance	63.0 (2.1)	59.0 (1.2)	61.2 (0.5)	64.0 (1.6)	<.001^c^		
	Swing	37.0 (2.1)	41.0 (1.2)	38.8 (0.5)	36.0 (1.6)	<.001^c^		
	Stance-to-swing	1.7 (0.2)	1.4 (1.2)	1.6 (0.03)	1.8 (0.1)	<.001^c^		

^a^The denominators of the percentages in these columns correspond to the n values in the “Total population” column of the same row.

^b^The *P* values are based on chi-square test for categorical variables and one-way ANOVA for numerical variables in the stance-to-swing ratio groups.

^c^ANOVA.

^d^Chi-square test.

### Cognitive Performance and Gait Balance

Table 2 shows the mean (SE) of each SNSB-C test (percentile) data according to the stance-to-swing ratios. The Digit Symbol Coding (56.31 vs 61.65 vs 52.88; *P*=.02), TMT-E: B (61.77 vs 57.76 vs 51.49; *P*=.02), and K-CWST: 60sec (45.67 vs 48.72 vs 40.87; *P*=.03) tests for frontal and executive functions showed statistically significant differences among the stance-to-swing ratio groups. However, there were no differences in the other cognitive functions.

**Table 2:** Mean (SE) of each Seoul Neuropsychological Screening Battery-Core (SNSB-C) test (percentile) according to the stance-to-swing ratio groups.

Function and test		Stance-to-swing ratio group, mean (SE)			*P* value^a^
		<1.50	1.50-1.63	>1.63	
**Attention**					
	Digit Span Test	44.25 (4.88)	50.22 (2.83)	43.71 (1.54)	.11
**Visuospatial**					
	RCFT^b^: copy	65.16 (4.37)	55.97 (2.44)	55.98 (1.53)	.19
**Language**					
	S-K-BNT^c^	53.41 (5.11)	58.57 (2.59)	53.35 (1.47)	.20
**Memory**					
	SVLT: DR^d^	38.64 (5.58)	32.95 (2.78)	36.77 (1.63)	.44
**Frontal and executive**					
	Digit Symbol Coding	56.31 (4.97)	61.65 (2.46)	52.88 (1.61)	.02
	COWAT^e^: animal + ㄱ	49.21 (4.88)	46.45 (2.61)	41.96 (1.50)	.16
	TMT-E: B^f^	61.77 (3.27)	57.76 (2.52)	51.49 (1.49)	.02
	K-CWST: 60sec^g^	45.67 (4.92)	48.72 (2.64)	40.87 (1.56)	.03

^a^The *P* values are based on one-way ANOVA for numerical variables in the stance-to-swing ratio groups.

^b^RCFT: Rey Complex Figure Test.

^c^S-K-BNT: short version of the Korean-Boston Naming Test.

^d^SVLT: DR: Seoul Verbal Learning Test: Delayed Recall.

^e^COWAT: Controlled Oral Word Association Test.

^f^TMT-E: B: Trail Making Test-Elderly: Part B.

^g^K-CWST: 60sec: the Korean version of Color Word Stroop Test: 60 seconds.

### Association Between Cognitive Performance and Gait Balance

Table 3 shows the estimated β coefficients (SE) of each SNSB-C test based on the stance-to-swing ratio groups. In the Digit Span Test for attention functions, a significant association was observed in the >1.63 ratio group (β=–6.514, SE 3.098; *P*=.04) before adjustment. The unadjusted β coefficients were significant in the >1.63 ratio group for the Digit Symbol Coding (β=–8.773, SE 3.098; *P*=.005), TMT-E: B (β=–6.269, SE 2.890; *P*=.03), and K-CWST: 60sec (β=–7.855, SE 3.075; *P*=.01) tests for frontal and executive functions. In the fully adjusted model, a significant β coefficient was observed in the >1.63 ratio group for the S-K-BNT for language function (β=–6.573, SE 2.813; *P*=.02). Compared with the reference, there were significant β coefficients in the >1.63 ratio group for the Digit Symbol Coding (β=–7.991, SE 3.028; *P*=.009) and K-CWST: 60sec (β=–8.083, SE 3.076; *P*=.009) tests for frontal and executive functions. In general, the association between the SNSB-C test data and the stance-to-swing ratio in the >1.63 ratio group yielded lower β coefficients than those in the 1.50-1.63 ratio group. Most β coefficients showed a decreasing relationship for the SNSB-C test data and the stance-to-swing ratio, whereas there were no significant associations in the <1.50 ratio group (all *P*>.05).

**Table 3:** The estimated β coefficient (SE) of each Seoul Neuropsychological Screening Battery-Core (SNSB-C) test based on the stance-to-swing ratio groups.

Function and test			Unadjusted model (stance-to-swing ratio group)											Adjusted^a^ model (stance-to-swing ratio group)								
			<1.50				1.50-1.63		>1.63				<1.50					1.50-1.63		>1.63		
			Value, β (SE)		*P* value				Value, β (SE)		*P* value		Value, β (SE)			*P* value				Value, β (SE)		*P* value
**Attention**																						
	Digit Span Test	–5.967 (5.723)		.30		Ref^b^		–6.514 (3.098)		.04		–5.474 (5.739)			.34		Ref		–4.141 (3.184)		.19	
**Visuospatial**																						
	RCFT^c^: copy	9.193 (5.461)		.09		Ref		0.014 (2.956)		>.99		8.913 (5.460)			.10		Ref		0.981 (3.029)		.75	
**Language**																						
	S-K-BNT^d^	–5.160 (5.444)		.34		Ref		–5.215 (2.947)		.08		–3.471 (5.071)			.49		Ref		–6.573 (2.813)		.02	
**Memory**																						
	SVLT: DR^e^	5.691 (5.979)		.34		Ref		3.817 (3.237)		.24		5.696 (5.724)			.32		Ref		3.479 (3.175)		.27	
**Frontal and executive**																						
	Digit Symbol Coding	–5.344 (5.706)		.35		Ref		–8.773 (3.089)		.005		–4.092 (5.458)			.45		Ref		–7.991 (3.028)		.009	
	COWAT^f^: animal + ㄱ	2.766 (5.514)		.62		Ref		–4.486 (2.985)		.13		2.904 (5.538)			.60		Ref		–5.175 (3.072)		.09	
	TMT-E: B^g^	4.016 (5.338)		.45		Ref		–6.269 (2.890)		.03		5.552 (5.290)			.29		Ref		–5.206 (2.934)		.08	
	K-CWST: 60sec^h^	–3.049 (5.680)		.59		Ref		–7.855 (3.075)		.01		–2.318 (5.545)			.68		Ref		–8.083 (3.076)		0=.009	

^a^This result was adjusted for age, sex, education, smoking status, alcohol consumption, vigorous exercises, and BMI.

^b^Ref: reference.

^c^RCFT: Rey Complex Figure Test.

^d^S-K-BNT: short version of the Korean-Boston Naming Test.

^e^SVLT: DR: Seoul Verbal Learning Test: Delayed Recall.

^f^COWAT: Controlled Oral Word Association Test.

^g^TMT-E: B: Trail Making Test-Elderly: Part B.

^h^K-CWST: 60sec: the Korean version of Color Word Stroop Test: 60seconds.

**Figure 1:**
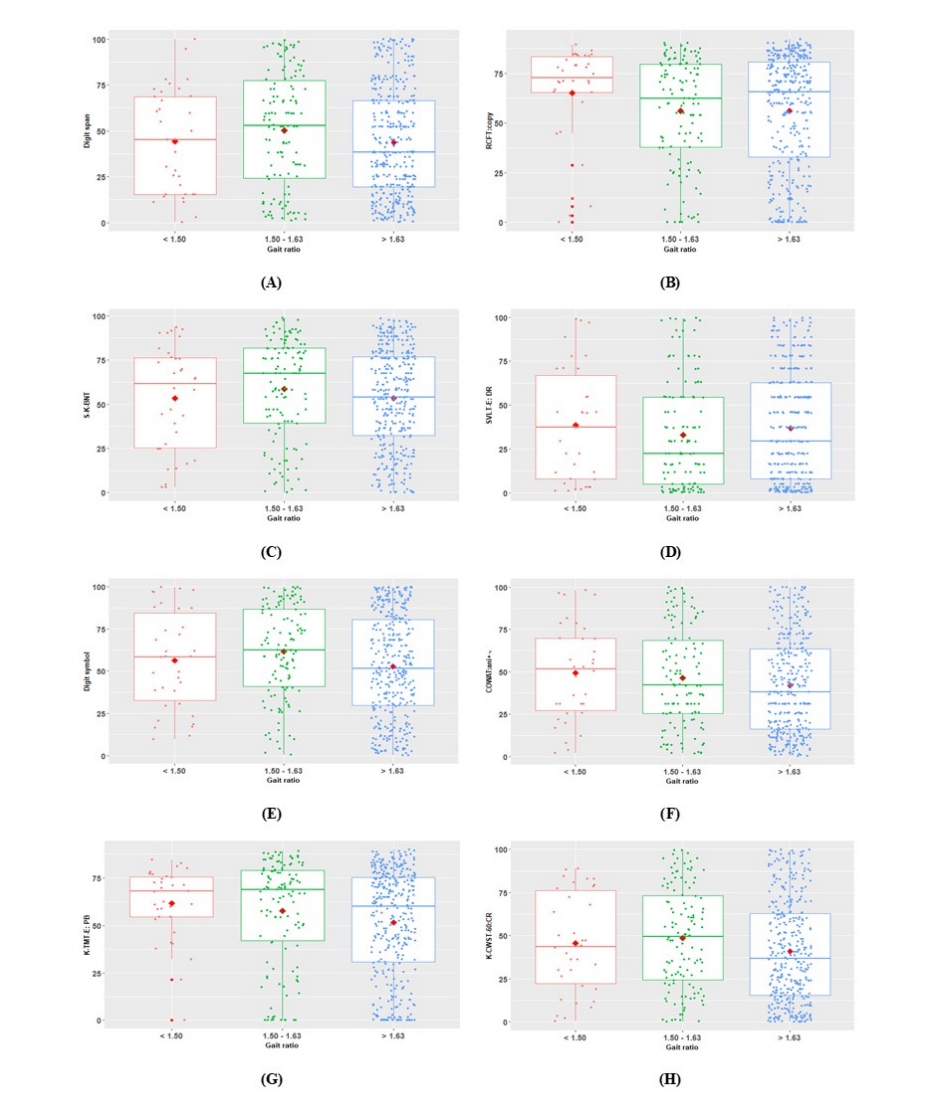
The box and scatter plots of the gait ratio groups for each SNSB-C test. The red box and scatter plots represent the <1.50 ratio group, the green box and scatter plots represent the 1.50-1.63 ratio group, and the blue box and scatter plots represent the >1.63 ratio group. The red dots are the mean of each SNSB-C test percentile according to each gait ratio group: (A) Digit Span Test, (B) RCFT: copy, (C) S-K-BNT, (D) SVLT: DR, (E) Digit Symbol Xoding, (F) COWAT: animal + ㄱ, (G) TMT-E: B, and (H) K-CWST: 60 sec. COWAT: Controlled Oral Word Association Test; K-CWST: 60sec: the Korean version of Color Word Stroop Test: 60 seconds; RCFT: Rey Complex Figure Test; S-K-BNT: short version of the Korean-Boston Naming Test; SNSB-C: Seoul Neuropsychological Screening Battery-Core; SVLT: DR: Seoul Verbal Learning Test: Delayed Recall; and TMT-E: B: Trail Making Test-Elderly: Part B.

Figure 1 presents the distributions between SNSB-C tests and stance-to-swing ratio groups using box and scatter plots. Overall, the study participants in the >1.63 gait ratio group were distributed across all the percentiles for each SNSB-C test.

**Figure 2:**
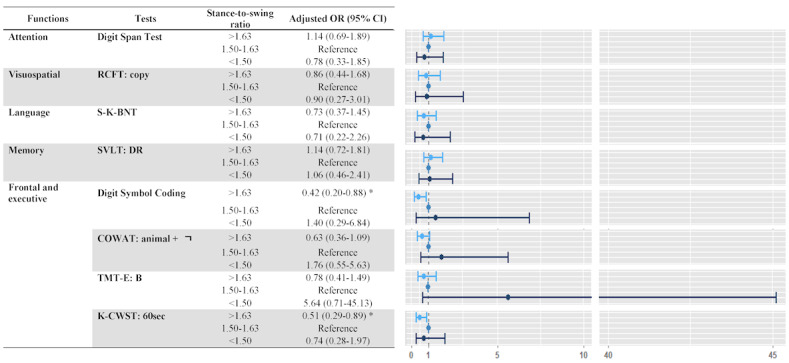
Adjusted odds ratio (OR; 95% CI) of each Seoul Neuropsychological Screening Battery-Core (SNSB-C) test according to the stance-to-swing ratio group. Adjusted for age, sex, education, smoking status, alcohol consumption, vigorous exercises, and BMI. *P<.001. COWAT: Controlled Oral Word Association Test; K-CWST: 60sec: the Korean version of Color Word Stroop Test: 60seconds; RCFT: Rey Complex Figure Test; S-K-BNT: short version of the Korean-Boston Naming Test; SVLT: DR: Seoul Verbal Learning Test: Delayed Recall; TMT-E: B: Trail Making Test-Elderly: Part B.

The adjusted OR (95% CI) for each SNSB-C test according to the stance-to-swing ratio group is shown in Figure 2. After adjustment for age, sex, education, smoking status, alcohol consumption, vigorous exercises, and BMI, the adjusted OR for the Digit Symbol Coding (adjusted OR 0.42, 95% CI 0.20-0.88) and K-CWST: 60sec (adjusted OR 0.51, 95% CI 0.29-0.89) tests for frontal and executive function was significantly lower for the >1.63 ratio group than for the reference group (the 1.50-1.63 stance-to-swing ratio group). However, most cognitive function tests showed no differences according to the stance-to-swing ratio group.

## Discussion

### Principal Findings

This study investigated the association between cognitive function and gait harmony in older Korean adults. We found a significantly higher cognitive performance percentile in frontal and executive functions among participants in the harmonic stance-to-swing ratio group. Compared with participants that exhibited a harmonic gait ratio, the cognitive assessment percentile decreased in those who showed the nonharmonic gait ratio, especially for visuospatial, frontal, and executive functions. Specifically, the relationship between the Digit Symbol Coding and K-CWST: 60sec test data on frontal and executive functions and the gait phase ratio remained significant after controlling for covariates. This information supports our understanding that cognitive function is interrelated with gait harmony in the older adult population.

### Comparison With Prior Work and Clinical Implications

Our findings on gait balance and cognitive function were partially consistent with the results of previous studies. According to a recent cross-sectional study by Noh et al [21] of 735 community-based individuals (aged 65-89 years), cognitive function was associated with the stance phase at a slower walking speed (β=0.088; *P*=.02). Based on a previous study in Italy on people with and without PD, alterations in gait ratio were expected; Peppe et al [27] suggested that compared with healthy participants, patients with PD have prolonged stance phases (patients with PD: 68.1% vs patients without PD: 63.6%; *P*<.001) [27]. Another study in 31 patients older than 55 years in China [46] investigated the relationship between gait characteristics and dementia, such as poststroke dementia (PSD) and AD. Ni et al [46] showed that in all gait tests, the percentage of time spent in the stance phase was longer (patients with PSD: 63.95% vs patients with AD: 63.09% vs healthy adults: 62.15%; *P*=.002) and in the swing phase was shorter (patients with PSD: 36.04% vs patients with AD: 36.91% vs healthy adults: 37.86%; *P*=.002) among patients with dementia than among healthy controls. Taken together, these studies highlight that older individuals with gait imbalance are relatively susceptible to a decrease in cognitive function.

The mechanism underlying the relationship between proper gait proportion and cognition remains unclear. One possible explanation is that a well-balanced gait is related not only to the motor system, such as muscle strength, but also to cognition, such as memory, attention, executive function, and visuospatial capacity [47,48]. Attention and executive functions are significantly associated with gait speed in older adults with and without cognitive decline [49]. Poor attention and executive processing in the brain are correlated with white matter hyperintensity, which negatively affects gait pace, spatiality, and variability [50]. The variability of gait is related to the stance and swing phases, and the double support phase is more variable in the presence of poor balance. In memory and spatial functions, beyond the primary role of the hippocampus and parahippocampal gyrus, the induction of memory by hippocampal atrophy also influences rhythm, variability, and human balance control [51-53]. Gait reflects the health of individuals in compensating for changes in postural balance and is controlled by well-balanced neural circuits and specific brain structures, including the frontal lobes, basal ganglia, cerebellum, and sensory and motor systems [48,54,55]. Generally, during walking, the complexity of controlling redundant degrees of freedom of bilateral multijoint limbs is reduced by the nervous system [56]. Human gait is structured for specific phases of the gait leg movements [57] that can lead to the maintenance of the basic walking rhythm, which generates alternating activity of the flexor and extensor motoneurons [58]. Simple alternation of flexor and extensor activity is converted into more complex and adaptable walking patterns by hippocampal neurons that receive serotonergic projections from the median raphe nuclei. The projections of serotonergic neurons of the brain stem that innervate the spinal cord stepping generator play a role in activating and influencing the walking rhythm [59]. A decrease in serotonin levels in cerebrospinal fluid and severe gait and posture disorders have been observed in patients with PD [60]. Hence, the stance-to-swing ratios under pathological conditions may be far from the value of the harmonic proportion value.

### Strengths and Limitations

To our knowledge, this study demonstrated an association between gait harmony and cognitive function, which has not been reported previously. Additionally, a strength of this study is the use of noninvasive measurement as an index for predicting decreased cognitive function. Moreover, we showed association between gait and cognitive performance using the SNSB-C tests, which includes 5 cognitive functions (ie, attention, visuospatial, language, memory, and frontal and executive). However, the study had some limitations. First, as this was a cross-sectional study, it is difficult to confirm the causal relationship between gait harmony and cognitive function. Therefore, we cannot indicate trends or changes from normal cognitive function to cognitive dysfunction. Second, the study was not free from bias due to self-reported data. As this study was based on an observational investigation, the questions asked to collect data may concern private or sensitive topics, such as education level, smoking status, and alcohol consumption. Thus, self-reporting data can be affected by social desirability. Moreover, we cannot rule out that some variables affecting cognition, such as dietary intake, medication, and occupation, were not analyzed in the statistical model. These unmeasured variables may be residual confounders.

### Conclusions

Dementia is one of the most important health issues associated with aging. However, the diagnosis of dementia is expensive and involves complex measurements. Gait, on the other hand, can be measured noninvasively, comparatively conveniently, and rapidly. Therefore, this study can be important for public health management, in that gait performance assessment can be used to screen for potential cognitive impairment.

Our study found that low cognitive function was related to a nonharmonic gait ratio in participants aged 60 years or older in South Korea. In contrast, harmonic gait ratio was associated with good cognitive performance. These findings suggest that the gait ratio may be a valuable indicator of cognitive impairment. The presence of a nonharmonic gait ratio that is different from the general gait ratio may be an indirect marker of cognitive decline, independent of confounding factors in the older adult population. However, more research is required to replicate our results and establish possible mechanisms of a relationship between proper gait ratio and cognitive function.

## Abbreviations

AD: Alzheimer disease
COWAT: Controlled Oral Word Association Test
IMU: inertial measurement unit
K-CWST: 60sec: the Korean version of Color Word Stroop Test: 60 seconds
OR: odds ratio
PD: Parkinson disease
PSD: poststroke dementia
RCFT: Rey Complex Figure Test
S-K-BNT: short version of the Korean-Boston Naming Test
SNSB-C: Seoul Neuropsychological Screening Battery-Core
SVLT DR: Seoul Verbal Learning Test: Delayed Recall
TMT-E B: Trail Making Test-Elderly: Part B
